# Ranking Predatory Journals: Solve the Problem Instead of Removing It!

**DOI:** 10.15171/apb.2016.001

**Published:** 2016-03-17

**Authors:** Mehdi Dadkhah, Giorgio Bianciardi

**Affiliations:** Department of Medical Biotechnologies, Anatomia Patologica, Siena University, 53100 Siena, Italy.

**Keywords:** Predatory journals, Ranking predatory journals, Journal selection, Academic ethic, Journal quality, Predatory journal criteria

## Abstract

Predatory journals are a well-known issue for scholarly publishing and they are repositories for bogus research. In recent years, the number of predatory journals has risen and it is necessary to present a solution for this challenge. In this paper, we will discuss about a possible ranking of predatory journals. Our ranking approach is based on Beall’s criteria for detection of predatory journals and it can help editors to improve their journals or convert their questionable journals to non-predatory ones. Moreover, our approach could help young editors to protect their journals against predatory practice. Finally, we present a case study to clarify our approach.

## Introduction

Nowadays, the academic world is faced with many challenges. Predatory journals are a well-known issue for scholarly publishing. These journals are based on “pay and publish” model and they are repositories for bogus research. Jeffrey Beall was the first one who introduced to the academic societies the term "predatory publishers". He started his research on questionable journals in early 2010. He gathered a list of 20 publishers and created his first predatory publisher list.^1^ In early 2014, his list contained about 700 predatory publishers and there was a fast growing for predatory journals.^2^ After Beall's research, some researchers and editors started to inspect that problem. They focused on the destructive effect of these journals on science and tried to present strategies for boycotting them.^3-6^ These papers acted to prevent the rapid growing of those predatory journals, but they were not able to stop them. Many researchers in developing countries haven’t any information about predatory journals and submit their papers to them. Moreover, some open access and indexed journals started to use predatory practice. Early predatory journals haven’t any indexing, but currently we can find some reputable indexed journals in Beall's predatory journal list.^7^

In this paper, we will discuss about a possible ranking of predatory journals. Our ranking approach is based on Beall’s criteria for detection of predatory journals and it can help editors to improve their journals or convert their questionable journals to non-predatory ones. Moreover, our approach could help young editors to protect their journals against predatory practice. In section 2, we introduce criteria for detection of predatory journals. In section 3, we present our approach for ranking predatory journals. Finally, we’ll present a case study to show how our approach can be used.

## Criteria for detecting predatory journals

We believe that is unfair to consider every predatory journal as equals (e.g., some predatory journals are only pure websites). We use Beall's criteria for determining a “degree of dangerousness” of them.^8^ We classify these criteria in four groups and expand them.

### Editorial member’s criteria

In most predatory journals, there aren’t any official emails related to the editor or they use general email services, such as Gmail.com or Yahoo.com. Affiliations of editors are not clearly written or they use Country name, only. Editors come from certain countries and the number of editors is very small. Some predatory journals use fake names or use names of standout researchers as editorial board members, without their permission. In the “contact section” of the journal website, there is only a form without any contact email or phone number.

### Review process and publishing

We underline that most predatory journals accept papers in a very fast manner (generally in one week) and they have unclear policies and review process. In each issue, we can find huge number of published papers. Some predatory journals publish questionable special issues in side of their regular issues. Where, a “questionable special issue” is an issue out of the journal scope and with the presence of many published papers. For example, a predatory biological journal creates a special issue and it publishes a lot of papers in the domains of computer hardware, civil engineering, etc.

### Announcements 

By inspecting predatory journals, we can find similar matter among them. In many cases, full address of the publisher is not available. They try to introduce themselves as an European- or American-based journal (such as “The American Journal of ...”). They use bogus metric to claim authors for submitting papers. We can recall: the “universal impact factor” (UIF), “global impact factor” (GIF) and the so-called “journal impact factor” (JIF) as popular bogus metrics of predatory journals.^9^ Some predatory journals use non-reputable indexing such as ISI (Indian science indexing) or fake citation reports.^10^ Spam emails are used by predatory journals in order to receive papers.^4^

### OA policies and publication charges

Predatory journals try to gain more money by presenting different charges such as fast track fee, publication charges, reader’s charges and submission charges. Some predatory journals charges both authors and readers; they request charges for publishing author’s papers and sell published papers to authors.

Publication charge is different than open access fee. In effect, while open access fee is an option in reputable journals, predatory journals have only publication charges.

## Predatory Rate: ranking predatory journals

We present a predatory ranking metric entitled "Predatory Rate", in order to rank the predatory journals. Table 1 shows criteria we propose to rank predatory journals (this metric is based on the above mentioned criteria, section 2).

**Table 1:** Criteria to rank predatory journals

**Criteria Group**	**Criteria**	**metric**	**weight**
Editorial section	Email of editor	Official email	0
		General email service	1
		Not available	2
	Affiliation of editors	Full affiliation	0
		Only country name	1
		Not available	2
		Editors are from certain country	2
	Number of editors	Lower than 5	2
		Between 5-7	1
		More than 7	0
Review process and publishing	Review time	Lower than a week	2
		Lower than a month	1
		More than a month	0
	Unclear review process	Yes	1
		No	0
	No. of paper in each issue	Lower than 20 papers	0
		More than 20 paper	1
	Questionable special issue	Yes	1
		No	0
announcement	Availability of journal full address	Yes	0
		No	1
	Using bogus metric and index	Yes	1
		No	0
	Send journal spam email to receive papers	Yes	1
		No	0
OA policies and publication charges	Fast track fee	Yes	1
		No	0
	Submission fee	Yes	1
		No	0
	Publication Fee	Yes	1
		No	0
	Charging both authors and readers	Yes	1
		No	0

To calculate the “Predatory rate”, in relation to those criteria, we use a weight that ranged between 1 and 3 for each criterion, then we sum all the values, dividing by n, where n is No of all criteria (1). No. of all criteria in our metric is equal to fourteen.

(1) $$PR=\frac{\sum weight}{n}$$

Predatory rate (PR) is a number between 0 and 1. If *PR* is equal to 0, it means that the journal is not a predatory one. If *PR* is higher than 0 and lower than 0.22, the journal uses predatory practices, in the other cases, the journals is predatory one, only. We obtained these values basing on our observations of 150 journals, 80 predatory and 70 non-predatory journals, 0.22 is the clustering value of the observed data. Based on *PR*, we can rank predatory journal. If a predatory journal wants to be converted to non-predatory journal, this journal has to go over all the criteria, trying to gain a value of PR close to 0.

This metric can be used by indexing centers able to evaluate journals for indexing or to detect predatory journals and dropping them, eventually. Editors could also use this metric to evaluate the *PR* index of their journals in order to prepare suitable policies for improving them. Unlike previous studies which point to boycott them, indiscriminately, we are trying to improve journals with a low level of predatory practice for a possible conversion to a complete non-predatory behavior. It is clear that journals with high predatory practices cannot be converted to non-predatory ones.

## A case study

To show our approach, we will evaluate three types of journals. We named these journals Case A, B and C. including predatory journal, journal with predatory practice and a non-predatory one. *Case A* and *B* are available in Beall’s predatory journal list. It means that these journals are predatory journals or journal with predatory practices. *Case C* is a science citation indexed one (Thomson-Reuters) and it is published by a University. Table 2 shows the criteria values for each journal. These values were determined by inspecting journals websites according to the guideline presented on section 2 and Table 1.

By using equation (1), we can calculate *PR* for these journals. Equations (2), (3) and (4) show calculated values.

(2) $${\text{PR}}_{\text{Case A}}\text{=}\frac{\text{1+1+1+1+1+1+1}}{\text{14}}\text{=0.5}$$

(3) $${\text{PR}}_{\text{Case B}}\text{=}\frac{\text{1+1+1}}{\text{14}}\text{=0.21}\;$$

(4) $${\text{PR}}_{\text{Case C}}\text{=}\frac{\text{0}}{\text{14}}\text{=0 }$$

According to calculated values, *Case A* is a predatory journal (PR > 0.22) and *Case B* is a journal with predatory practices (PR < 0.22 and PR > 0). Mentioned journals are available in Beall’s list of predatory journals, thus confirming our approach. *PR* is equal to 0 for *Case C*, thus it is a non-predatory journal.

**Table 2:** Value of criteria for each journals in the case study

**Criteria Group**	**Criteria**	**metric**	**weight**	**Case A**	**Case B**	**Case C**
**Editorial section**	Email of editor	Official email	0	-	×	×
		General email service	1	×	-	-
		Not available	2	-	-	-
	Affiliation of editors	Full affiliation	0	×	×	×
		Only country name	1	-	-	-
		Not available	2	-	-	-
		Editors are from certain country	2	-	-	-
	Number of editors	Lower than 5	2	-	-	-
		Between 5-7	1	-	-	-
		More than 7	0	×	×	×
**Review process and publishing**	Review time	Lower than a week	2	-	-	-
		Lower than a month	1	×	×	-
		More than a month	0	-	-	×
	Unclear review process	Yes	1	×	-	-
		No	0	-	×	×
	No. of paper in each issue	Lower than 20 papers	0	×	-	×
		More than 20 paper	1	-	×	-
	Questionable special issue	Yes	1	-	-	-
		No	0	×	×	×
**announcement**	Availability of journal full address	Yes	0	-	×	×
		No	1	×	-	-
	Using bogus metric and index	Yes	1	×	-	-
		No	0	-	×	×
	Send journal spam email to receive papers	Yes	1	×	-	-
		No	0	-	×	×
**OA policies and publication charges**	Fast track fee	Yes	1	-	-	-
		No	0	×	×	×
	Submission fee	Yes	1	-	-	-
		No	0	×	×	×
	Publication Fee	Yes	1	×	×	
		No	0	-	-	×
	Charging authors and readers, both	Yes	1	-	-	-
		No	0	×	×	×

## Conclusion

In this paper, we introduce a new metric, the Predatory Rate, PR, for ranking journals. This metric helps us to do judgment about predatory journals and let editors to evaluate their journals against predatory practices. Academic databases could use this metric to indicate the journal predatory rate in their evaluation process. According this metric, journals would be classified in three groups as follows: predatory journals, journal with predatory practice, and non-predatory ones, also in order to help a journal with predatory practice to be converted to a non-predatory one.

## Ethical Issues

Not applicable.

## Conflict of Interest

The authors report no conflicts of interest.
